# Differentiation of uterine low-grade endometrial stromal sarcoma from rare leiomyoma variants by magnetic resonance imaging

**DOI:** 10.1038/s41598-021-98473-z

**Published:** 2021-09-27

**Authors:** Yuki Himoto, Aki Kido, Akihiko Sakata, Yusaku Moribata, Yasuhisa Kurata, Ayako Suzuki, Noriomi Matsumura, Fuki Shitano, Seiya Kawahara, Shigeto Kubo, Shigeaki Umeoka, Sachiko Minamiguchi, Masaki Mandai

**Affiliations:** 1 Department of Diagnostic Radiology and Nuclear Medicine, Kyoto University Hospital, 54 Kawahara-cho, Shogoin, Sakyoku, Kyoto, 606-8507 Japan; 2 Department of Obstetrics and Gynecology, Kindai University Faculty of Medicine, Osaka, Japan; 3 Department of Radiology, Japanese Red Cross Osaka Hospital, Osaka, Japan; 4 Department of Radiology, Japanese Red Cross Otsu Hospital, Shiga, Japan; 5 Department of Radiology, Kitano Hospital, Osaka, Japan; 6 Department of Diagnostic Radiology, Japanese Red Cross Wakayama Medical Center, Wakayama, Japan; 7 Department of Diagnostic Pathology, Kyoto University, Kyoto, Japan; 8 Department of Gynecology and Obstetrics, Kyoto University, Kyoto, Japan

**Keywords:** Gynaecological cancer, Diagnosis, Magnetic resonance imaging

## Abstract

The purpose of this study is to evaluate utility of MRI in differentiation of uterine low-grade endometrial stromal sarcoma (LGESS) from rare leiomyoma variants. This multi-center retrospective study included consecutive 25 patients with uterine LGESS and 42 patients with rare leiomyoma variants who had pretreatment MRI. Two radiologists (R1/R2) independently evaluated MRI features, which were analyzed statistically using Fisher’s exact test or Student's *t*-test. Subsequently, using a five-point Likert scale, the two radiologists evaluated the diagnostic performance of a pre-defined MRI system using features reported as characteristics of LGESS in previous case series: uterine tumor with high signal intensity (SI) on diffusion-weighted images and with either worm-like nodular extension, intra-tumoral low SI bands, or low SI rim on T2-weighted images. Area under the receiver operating characteristic curve (AUC), sensitivity, and specificity of the two readers’ Likert scales were analyzed. Intra-tumoral low SI bands (*p* < 0.001), cystic/necrotic change (*p* ≤ 0.02), absence of speckled appearance (*p* < 0.001) on T2-weighted images, and a low apparent diffusion coefficient value (*p* ≤ 0.02) were significantly associated with LGESS. The pre-defined MRI system showed very good diagnostic performance: AUC 0.86/0.89, sensitivity 0.95/0.95, and specificity 0.67/0.69 for R1/R2. MRI can be useful to differentiate uterine LGESS from rare leiomyoma variants.

## Introduction

Endometrial stromal sarcoma (ESS), a very rare uterine sarcoma entity composed of tumor cells resembling endometrial stromal cells, constitutes 7–25% of uterine sarcomas, second to leiomyosarcoma, but accounting for only 0.2% of uterine malignancies (annual incidence, 0.19 per 100,000 women)^1^. Low-grade ESS (LGESS), a less aggressive subtype of ESS, typically develops in premenopausal and perimenopausal women^1,2^. Although it is indolent compared to high-grade ESS and leiomyosarcoma, LGESS frequently shows malignant behavior, including extra-uterine involvement, metastasis, and recurrence^1,2^.

Differential considerations in the diagnosis of LGESS include leiomyoma, adenomyosis, endometrial cancer, and other uterine sarcomas. Among them, pretreatment differentiation from benign lesions and recognition of its malignancy is the most clinically relevant issue^3–5^. Because of its rarity, nonspecific symptoms and preponderance among young women, LGESS can be misdiagnosed as leiomyoma or adenomyosis. It can lead to less-invasive treatment options for presumed benign indications such as enucleation with/without power morcellation, instead of hysterectomy and bilateral salpingo-oophorectomy as a standard treatment^5,6^. Since the US Food and Drug Administration issued a warning against its use in 2014, the safety of power morcellation has been debated, weighing patients’ benefits of minimal invasiveness and risks of occult cancer^7^. Reportedly, LGESS is a dominant type of unexpected uterine sarcomas after laparoscopic power morcellation^4^. It can be an unfavorable factor by increasing intraperitoneal spreading^1,8^. To perform minimally invasive procedures for leiomyoma safely, further improvement in pretreatment workup to detect LGESS is mandatory^8^.

Pretreatment differentiation of uterine sarcoma from leiomyoma by magnetic resonance imaging (MRI) has persisted as a challenge. Most leiomyomas with typical MRI appearance are easy to diagnose, but some rare leiomyoma variants might mimic sarcoma^9^. Regarding leiomyosarcoma, evidence has been accumulated supporting the utility of MRI. Lakhman et al. reported a classification model with high sensitivity and specificity using conventional MRI features to differentiate leiomyosarcoma from benign rare leiomyoma variants^10^. Recently, diffusion-weighted imaging (DWI) has been highlighted as a potentially useful tool for the differentiation of uterine sarcoma, reflecting the cellular density of tumor^11–13^. Compared to leiomyosarcoma, evidence of MRI about LGESS remains very limited because of its extreme rarity^5,14^. Characteristic MRI features of LGESS have been reported in small case series: worm-like nodular extension^15^, intra-tumoral low signal intensity (SI) bands on T2 weighted-images (T2WI)^16^, and low SI rim on T2WI^17^. Li et al. reported that 13 of 14 patients (93%) showed high SI on DWI^18^. Nevertheless, no report of the relevant literature describes the diagnostic performance of MRI in differentiation from leiomyoma, especially from rare leiomyoma variants.

The initial purpose of this multi-center study was to assess different MRI features on LGESS and leiomyoma variants. The second purpose was to analyze if a pre-defined MRI system using features reported previously as a characteristic of LGESS could be used for differential diagnosis with leiomyoma variants.

## Methods

This multi-center retrospective Health Insurance Portability and Accountability Act-compliant study was approved and the need for informed consent was waived by the respective institutional review boards of seven institutions: Kyoto University Hospital, Kindai University Faculty of Medicine, Kobe City Medical Center General Hospital, Japanese Red Cross Osaka Hospital, Japanese Red Cross Otsu Hospital, Kitano Hospital, and Japanese Red Cross Wakayama Medical Center. All methods were carried out in accordance with relevant guidelines and regulations.

### Patients

We searched each institutional pathological database consecutively for patients who were histologically diagnosed as having LGESS based on surgical specimen or biopsy and who had pretreatment MRI. Between January 2007 and June 2019, 25 patients from seven institutions were identified (LGESS group).

We also searched pathological databases of two institutions consecutively for patients who were histologically diagnosed as having cellular leiomyoma, leiomyoma with bizarre nuclei or atypical leiomyoma, mitotically active leiomyoma, or intravenous leiomyomatosis based on surgical specimens, and who had undergone pretreatment MRI. These variants were selected, referring a previous paper about the differentiation of leiomyosarcoma from leiomyoma variants^10^ and focusing on the variants with high cellularity/ cytologic atypia / mitotic activity. Smooth muscle tumor of uncertain malignant potential was not included because of its uncertain malignancy. During the same period, 42 patients were identified, constituting the Rare leiomyoma variant group.

All cases were diagnosed histologically by board-certified pathologists of the respective institutions, according to the World Health Organization’s Classification of Tumours of Female Reproductive Organs 2014^19^. Patient enrollments and excluded cases of the two groups are described in Supplemental Fig. 1. Pretreatment diagnosis, operative methods, intervals between MRI and surgery/biopsy, intervals between surgery/biopsy and the last follow-up, and patient status were reviewed from medical charts.

**Figure 1:**
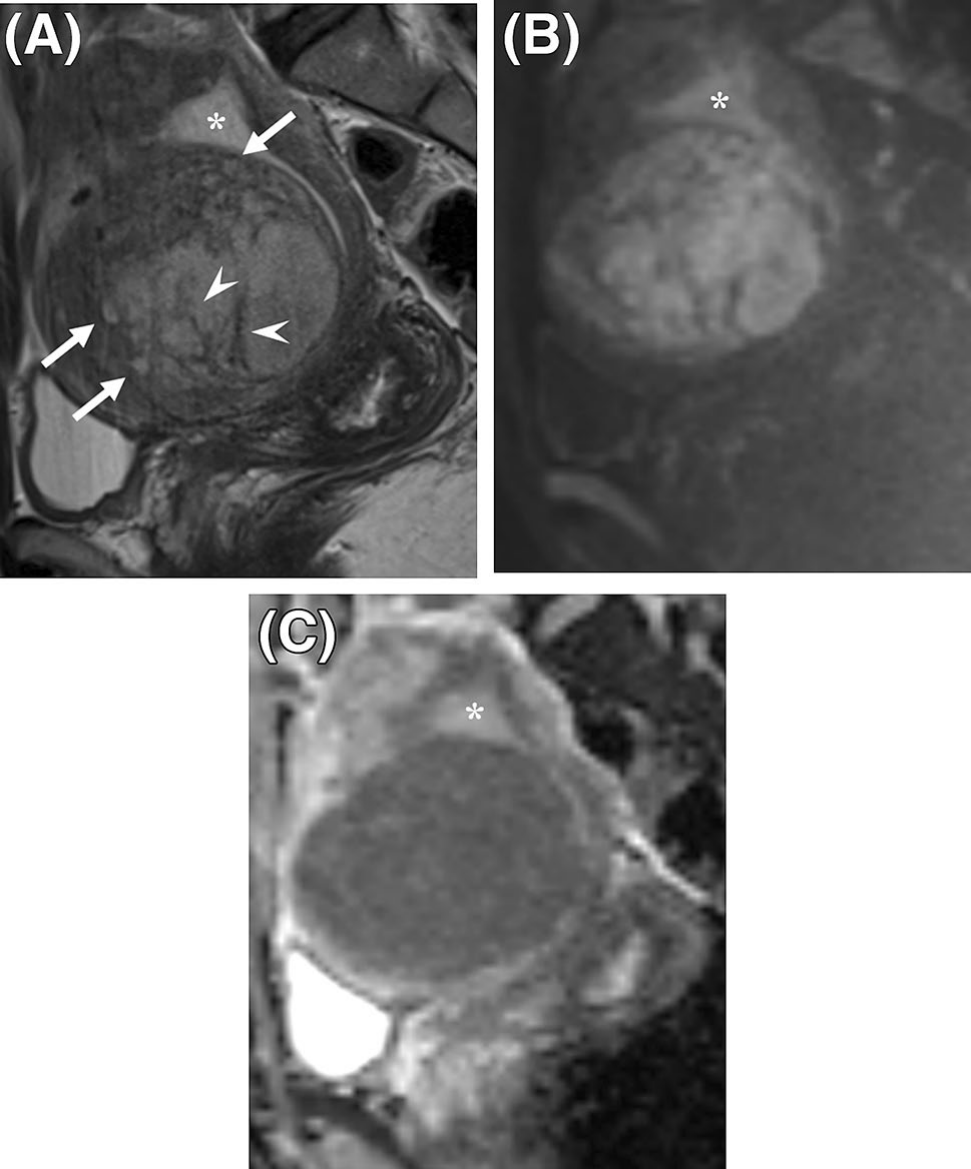
A representative case of low grade endometrial stromal sarcoma, showing worm-like nodular extension (**A**, arrows) and intra-tumoral low signal intensity (SI) bands (**A**, arrowheads) on T2-weighted images. The tumor shows high SI on diffusion-weighted images (**B**) with low apparent diffusion coefficient values (**C**). Asterisks were placed on endometrium (**A–C**).

### MRI protocols

All MRI studies were acquired in supine position on 1.5 T or 3.0 T scanners using pelvic phased-array coils. At a minimum, each study included two planes of T2WI (axial/coronal/sagittal), axial or sagittal T1-weighted images (T1WI) with/without fat saturation. Twenty-two patients of the LGESS group and all patients of the Rare leiomyoma variant group had undergone DWI. The high b values of participating institutions were 800–1000 s/mm^2^. Apparent diffusion coefficient (ADC) maps generated from DWI were available for 18 cases of the LGESS group and for 37 cases of the Rare leiomyoma variant group. Twenty-two cases of the LGESS group and 21 cases of the Rare leiomyoma variant group had contrast enhanced T1-weighted images (CE-T1WI) with/without fat saturation after administration of 0.1 or 0.2 mmol/kg gadolinium chelate contrast medium. Representative MRI protocols at the respective institutions are presented in Supplemental Table 1.

### Qualitative MRI features

**Figure 2:**
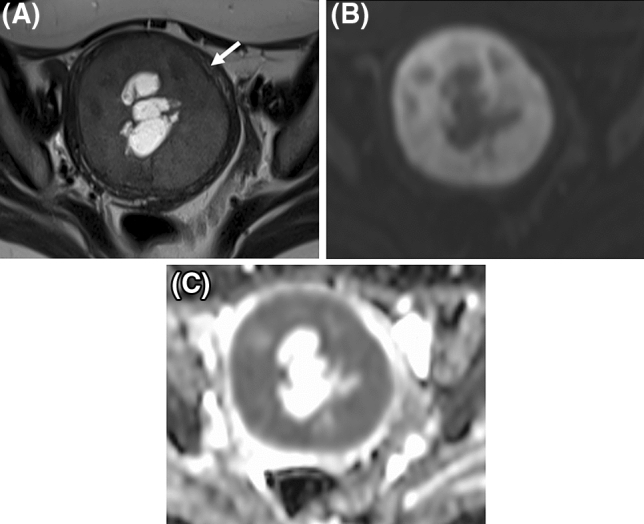
A representative case of low grade endometrial stromal sarcoma with surrounding low signal intensity (SI) rim on T2-weighted images (**A**, arrow). Cystic/necrotic change is also observed. Solid parts of tumor shows high SI on diffusion-weighted images (**B**) with low apparent diffusion coefficient values (**C**).

**Figure 3:**
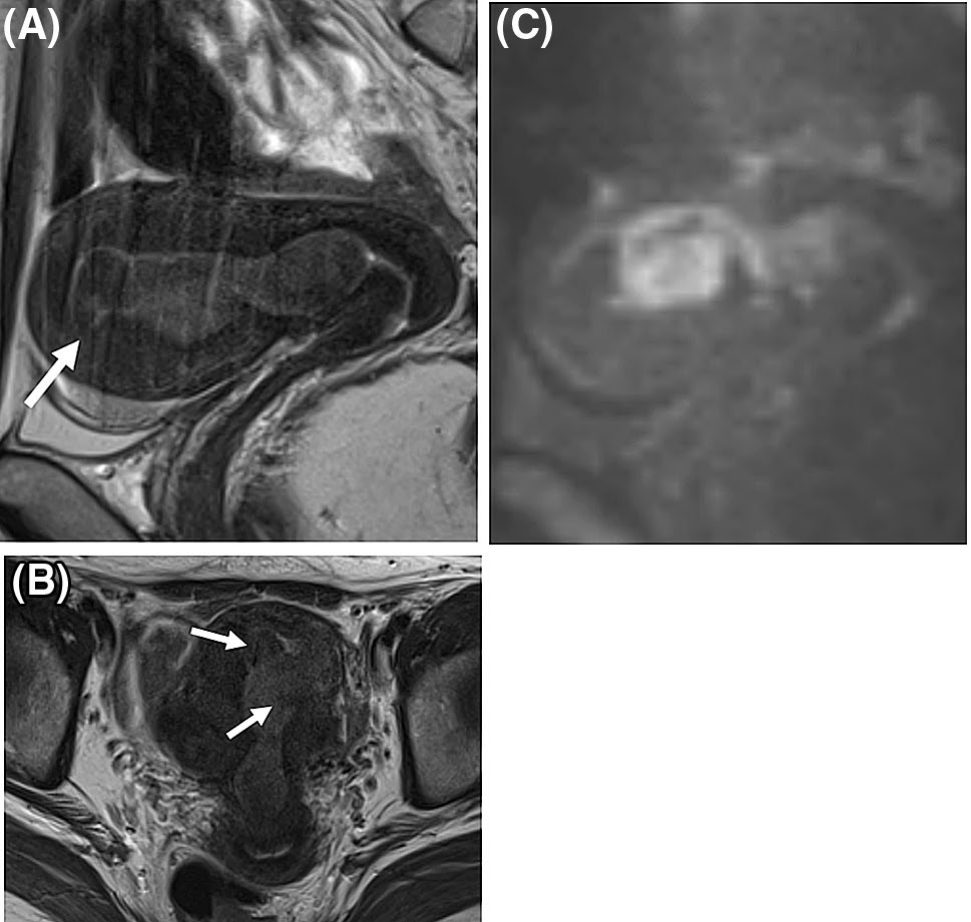
A false-negative case of low-grade endometrial stromal sarcoma. Tumor predominantly located in endometrial cavity with a small portion of myometrial invasion (**A**, arrow) on T2-weighted images. There might be intra-tumoral low signal intensity (SI) bands on T2-weighted images (**B**, arrows), but unclear. The tumor showed high SI on diffusion-weighted images (**C**) with low apparent diffusion coefficient values.

Two board-certified radiologists with 11 years of experience (R1, subspecialty in gynecologic imaging; R2, subspecialty in neuroimaging) independently reviewed pretreatment MRI, after trained to identify MRI features of LGESS with previous reports^9,10,15–17,20^. They were aware that patients had been diagnosed histologically as having either LGESS or rare leiomyoma variants, but they were blinded to all other clinical information. The following qualitative features were evaluated: on T2WI, (1) main site of the tumor (intramural, submucosal, or subserosal), (2) nodular and/or irregular border^15^, (3) extrauterine involvement, (4) worm-like nodular extension^15^, (5) intra-tumoral low SI bands^16^, (6) low SI rim surrounding intra-myometrial tumor^17^, (7) speckled appearance (reported to be characteristic of leiomyoma)^9,20^, (8) cystic and/or necrotic change^18^, (9) higher SI than outer myometrium; on T1WI images, (10) tumoral hemorrhage (intra-tumoral high SI), with worm-like nodular extension on T2WI defined as detached nodules from the primary tumor^15^, with speckled appearance defined as diffusely scattered high SI within the tumor on T2WI^20^. When DWI and CE-T1WI were available, the following features were assessed: (11) SI relative to sciatic nerve roots (iso or high vs low) on DWI and (12) heterogeneous tumor enhancement on CE-T1WI. Case examples of respective qualitative features were in Figs. 1, 2 and 3 and Supplemental Figs. 2, 3 and 4.

**Table 1:** Patient characteristics.

	LGESS (N = 25)	Rare leiomyoma variants (N = 42)
Age (year), median (range)	47 (20—78)	45.5 (22—67)
**Menopausal status**		
Premenopausal	14	37
Postmenopausal	11	5
**Treatment**		
Total hystrectomy	22	31
Enucleation	2	9
Excision	0	2
Chemotherapy	1	0
**Invasion beyond uterus**	4^a^	1
Lung	1	NA
Bone	1	NA
Ovary	1	NA
Peritoneum	2	NA
Ovarian vein	0	1
**Prognosis**		
No recurrence	22	42
Recurrence	2	0
Death	1	0
Follow up intervals (month), median (range)	61 (7—147)	23 (1—103)

*LGESS* low grade endometrial stromal sarcoma.

^a^One case had ovarian metastasis and peritoneal dissemination.

**Table 2:** Two readers' evaluations of magnetic resonance imaging features.

**T2-weighted image**	Reader 1			Reader 2		
	LGESS	Rare leiomyoma variants	*p* value	LGESS	Rare leiomyoma variants	*p* value
	N = 25	N = 42		N = 25	N = 42	
Main site						
Intramural	22 (88%)	31 (74%)		22 (88%)	38 (90%)	
Submucosal	2 (8%)	3 (7%)		2 (8%)	0 (0%)	
Subserosal	1 (4%)	8 (19%)		1 (4%)	4 (10%)	
Tumor size (mm, median, range)	85, 37–159	73, 12–172	0.14	84, 36–151	69, 7–191	0.14
Nodular and/or irregular border	13 (52%)	16 (64%)	0.31	18 (72%)	14 (33%)	< 0.01
Extrauterine involvement	3 (12%)	1 (2%)	0.14	2 (8%)	2 (8%)	0.62
Worm-like nodular extension	10 (40%)	8 (19%)	0.09	16 (64%)	8 (19%)	< 0.001
Intra-tumoral low SI bands*	19 (76%)	8 (19%)	< 0.001	19 (76%)	9 (21%)	< 0.001
Low SI rim	12 (48%)	10 (40%)	0.06	17 (68%)	7 (17%)	< 0.001
Speckled appearance*	3 (12%)	35 (83%)	< 0.001	3 (12%)	26 (62%)	< 0.001
Cystic and/or necrotic change*	17 (68%)	14 (33%)	0.01	14 (56%)	11 (26%)	0.02
Higher SI than outer myometrium	2 (8%)	3 (12%)	1.00	3 (12%)	9 (21%)	0.51
**T1-weighted image**						
Tumoral hemorrhage	10 (40%)	6 (14%)	0.04	4 (16%)	2 (5%)	0.19
**Diffusion-weighted image**	N = 22	N = 42		N = 22	N = 42	
SI higher than or equal to sciatic nerve	22 (100%)	37 (88%)	0.15	22 (100%)	38 (90%)	0.29
**ADC map**	N = 18	N = 37		N = 18	N = 37	
ADC value (× 10^-6^mm^2^/s, median, range)*	848, 443–1152	1087,230–1750	< 0.01	698, 258–1034	837, 456–1709	0.02
Normalized ADC value (median, range)*	0.29, 0.14–0.43	0.37, 0.10–0.68	0.001	0.24, 0.08–0.37	0.30, 0.17–0.53	0.001
**Contrast enhanced T1-weighted image**	N = 17	N = 18		N = 17	N = 18	
Heterogeneous tumor enhancement	12 (70%)	8 (44%)	0.18	10 (59%)	11 (61%)	1.00

*LGESS* low grade endometrial stromal sarcoma, *SI* signal intensity, *ADC* apparent diffusion coefficient.

*p value < 0.05 for both readers.

**Figure 4:**
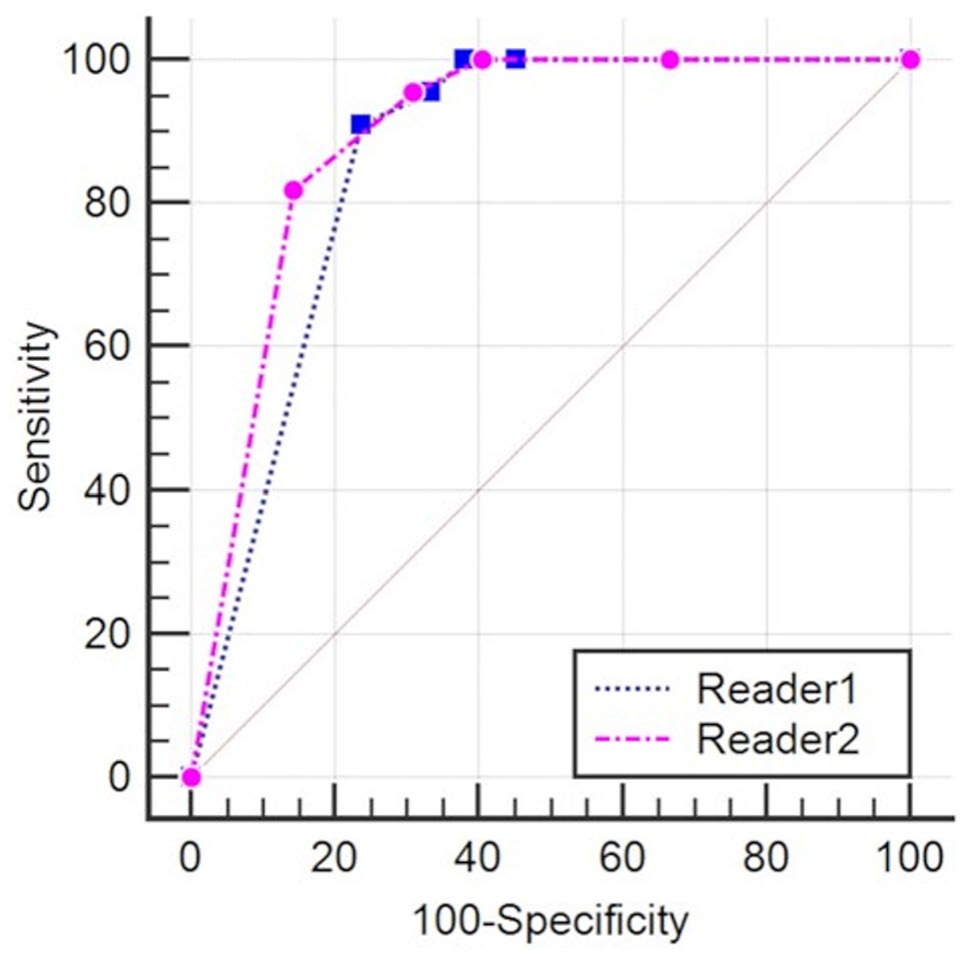
Receiver operating characteristic curves of the pre-defined MRI system for two readers in differentiation of low-grade endometrial stromal sarcoma from rare leiomyoma variants.

**Table 3:** Diagnostic performance of the pre-defined MRI system in differentiation of low grade endometrial stromal sarcoma from rare leiomyoma variants.

	Reader 1	Reader 2
AUC (SE, 95% CI)	0.86 (0.038, 0.75–0.94)	0.89 (0.036, 0.79–0.96)
Sensitivity	95%	95%
Specificity	67%	69%
PPV	60%	62%
NPV	97%	97%
Accuracy	77%	78%
Weighted Kappa of Likert scales (SE, 95% CI)	0.59 (0.069, 0.46–0.73)	
Kappa after dichotomization of Likert scales^a^ (SE, 95% CI)	0.78 (0.078, 0.63–0.93)	

*AUC* area under the curve, *SE* standard error, *CI* confidence interval, *PPV* positive predictive value, *NPV* negative predictive value.

^a^Cutoff of Likert scales, > 3.

### Quantitative MRI features

The two readers also evaluated the following quantitative features independently. The maximum tumor size was measured on either the sagittal, axial, or coronal T2WI plane. When an ADC map was available, referring other MRI sequences, three circular regions of interest (more than 25 mm^2^) were placed manually on solid parts of the tumor, carefully avoiding cystic/necrotic/hemorrhagic areas. Then, the ADC value of the tumor was measured as the mean value of three circular regions of interest. The normalized ADC value (the ADC value of tumor divided by the ADC value of urine) was also calculated to reduce the effect of ADC variation from mixed DWI protocols^21^.

### Pre-defined MRI system for differentiation of LGESS from leiomyoma

A pre-defined MRI system was created before evaluations of qualitative and quantitative MRI features above to avoid testimation (estimation after testing) bias. This system was based on T2WI and DWI features reported previously as a characteristic of LGESS: tumor with iso/high SI on DWI^18^ relative to sciatic nerve roots and with either worm-like nodular extension^15^, intra-tumoral T2 low SI bands^16^, or T2 low SI rim^17^. Among patients with DWI (22 of LGESS group and all of Rare leiomyoma variant group), the two readers (R1/R2) used a Likert scale for independent evaluation of whether the uterine tumor met the criteria, or not. Its scales were 1–5: 1, definitely no; 2, probably no; 3, equivocal; 4, probably yes; 5, definitely yes. Before the evaluations, two readers were informed that scales of 4 and 5 would be regarded positive, whereas scales of 1–3 negative, to calculate diagnostic performance, such as sensitivity and specificity.

### Statistical analysis

The associations between MRI features and LGESS were analyzed using Fisher’s exact test for qualitative data and Student *t*-test for quantitative data. Concordance correlation coefficients (CCCs) were calculated for quantitative features between R1 and R2: maximum tumor diameter, the ADC value, and the normalized ADC value. Regarding CCC values, the following were inferred: greater than 0.99 were considered almost perfect; > 0.95 to ≤ 0.99, substantial; > 0.90 to ≤ 0.95, moderate; and ≤ 0.90, poor^22^.

Receiver operating characteristic (ROC) curve analysis of the pre-defined MRI system was applied for each reader’s Likert scales. Area under the curve (AUC) with its standard error calculated using the DeLong method were computed: AUC greater than 0.90 was considered excellent diagnostic accuracy; 0.81–0.90, very good; 0.71–0.80, good; 0.61–0.70, moderate; 0.51–0.60, poor; ≤ 0.5, test not useful^23^. Then Likert scales were dichotomized with scales of 1–3 indicating leiomyoma and scales of 4 and 5 indicating LGESS to calculate sensitivity, specificity, positive predictive value (PPV), negative predictive value (NPV), and accuracy of the MRI criteria for R1/R2.

Reader agreement on the Likert scales of the pre-defined MRI system was analyzed using a weighted kappa statistic: *κ* > 0.80, excellent agreement; 0.61 < *κ* ≤ 0.80, good agreement; 0.41 < *κ* ≤ 0.60, moderate agreement; 0.21 < *κ* ≤ 0.40, fair; *κ* ≤ 0.20, poor agreement^24^. Reader agreement after dichotomization of Likert scales (cutoff, > 3) was also calculated for the pre-defined MRI system.

Statistical analyses were performed using software (Medcalc^®^ version 18.5; MedCalc Software, Ostend, Belgium). For all statistical analyses, a *p* value result of less than 0.05 was inferred as significant.

## Results

### Patients

Patient characteristics of the LGESS group and Rare leiomyoma variant group are presented in Table 1. The intervals between pretreatment MRI and surgery/biopsy were 3–291 days (median, 50 days) in the LGESS group and 1–604 days (median, 34 days) in the Rare leiomyoma variant group.

In the LGESS group, a malignant tumor was suspected before treatment in 15 cases (60%; sarcoma, *n* = 14; endometrial cancer, *n* = 1), whereas benign disease was presumed preoperatively in ten cases (40%; leiomyoma, *n* = 9; adenomyosis, *n* = 1). All patients were diagnosed as having LGESS by surgical specimen, except for one patient diagnosed by biopsy, who was not regarded as a surgery candidate because of multiple bone metastases.

The Rare leiomyoma variant group included 25 cases of cellular leiomyoma (preoperative diagnosis: leiomyoma, n = 15; degenerated leiomyoma, n = 6; adenomatoid tumor, n = 1; sarcoma, n = 2; cervical cancer, n = 1), nine cases of leiomyoma with bizarre nuclei (preoperative diagnosis: leiomyoma, n = 8; degenerated leiomyoma, n = 1), two cases of atypical leiomyoma (preoperative diagnosis: leiomyoma, n = 2), four cases of mitotically active leiomyoma (preoperative diagnosis: leiomyoma, n = 2; cellular leiomyoma, n = 1; sarcoma, n = 1), and two cases of intravenous leiomyomatosis (preoperative diagnosis: intravenous leiomyomatosis, n = 2). All were diagnosed by surgical specimen.

### MRI features

The two readers’ evaluations of MRI features are presented in Table 2. Among the qualitative features, intra-tumoral low SI bands, cystic/necrotic change, and absence of speckled appearance on T2WI were associated significantly with LGESS in both readers’ evaluations. Sensitivity/specificity for R1 and R2 were as follows: intra-tumoral low SI bands, R1 76%/81% and R2 76%/79%; cystic/necrotic change, R1 68%/67% and R2 56%/74%; absence of speckled appearance, R1 88%/83% and R2 88%/62%. All LGESS cases showed high/iso SI to sciatic nerve on DWI, although; most of the rare leiomyoma variants also did (sensitivity/specificity: R1, 100%/12%; R2, 100%/10%). Representative cases of LGESS are shown in Figs. 1 and 2.

Regarding quantitative MRI features, an ADC value (*p* value, < 0.01/0.02 for R1/R2; CCC, 0.51, poor; optimal cutoff, ≤ 932 10^–6^ mm^2^/s / ≤ 776 10^–6^ mm^2^/s for R1/R2) and a normalized ADC value (*p* value, 0.001 for both readers; CCC, 0.47, poor; optimal cutoff, ≤ 0.31/ ≤ 0.27 for R1/R2) of LGESS were lower than those of rare leiomyoma variants for both readers. With respective optimal cutoffs, sensitivity/specificity for R1 and R2 were as follows: the ADC value, R1 78%/76% and R2 89%/65%; the normalized ADC value, R1 72%/78% and R2 89%/70%. No significant association was found for the maximum tumor size (*p* value, 0.14 for both readers; CCC, 0.93, substantial).

### Pre-defined MRI system for differentiation of LGESS from leiomyoma

The diagnostic performance of the pre-defined MRI system was very good for R1 (AUC, 0.86) and R2 (AUC, 0.89). With the pre-defined cutoff > 3 of a Likert scale, the system showed high sensitivity (95% for both readers) and relatively low specificity (67% and 69%). One LGESS case was diagnosed as leiomyoma with a scale of 3 assigned by both readers (false negative), which was a tumor located predominantly in the endometrial cavity with a small portion of myometrial invasion (Fig. 3). Regarding false positive results, 14/13 cases of rare leiomyoma variants were diagnosed LGESS, respectively, by R1/R2. Two readers’ evaluations of MRI features for false-positive cases are in Supplemental Table 2 (Supplemental Fig. 3 and 4, representative false-positive cases of cellular leiomyoma). Figure 4 portrays ROC curves of the pre-defined MRI system based on the two readers’ evaluations. The inter-rater agreement of Likert scales between the two readers was moderate (*κ* = 0.59). After dichotomizing the Likert scale (1–3 vs 4 & 5), the inter-rater agreement was improved to be good (κ = 0.78). Details are presented in Table 3.

## Discussion

This multicenter retrospective study revealed specific MRI features that are significantly associated with LGESS, with comparison to rare leiomyoma variants. We also evaluated the diagnostic performance of the pre-defined MRI system for LGESS based on earlier reported MRI features. The diagnostic system showed very good diagnostic performance with high sensitivity (AUC 0.86/0.89, sensitivity 0.95/0.95, specificity 0.67/0.69 for R1/R2). A non-expert radiologist in gynecologic imaging obtained an AUC as good as a specialized radiologist. Our results suggest the ability of MRI to differentiate LGESS from rare leiomyoma variants and to optimize treatment selection. This MRI system would be clinically applicable to screen out LGESS in pretreatment workup for cases with higher suspicion of LGESS or leiomyomas with unusual appearances on MRI.

Three qualitative T2WI features were significantly associated with LGESS in both readers’ evaluations, including intra-tumoral low SI bands (considered to represent bundles of preserved myometrial fibers^16^, Fig. 1), cystic/necrotic change (Fig. 2), and absence of speckled appearance (a characteristic feature of leiomyoma^9,20^, Supplemental Fig. 3). Morphologically rich variations of LGESS on MRI and their correspondent T2WI features have been reported: poorly-demarcated tumor infiltrating to myometrium with worm-like nodular extension^15^ and/or intra-tumoral low SI bands (Fig. 1A)^16^; well-demarcated expansile myometrial mass with cystic degeneration and surrounding low SI rim (Fig. 2A)^17^; and tumor predominantly located in endometrial cavity^16^. However, because of the rarity and rich variations, it has been difficult to grasp the whole MRI image of LGESS from small case series. The three qualitative T2WI features would be key findings to suspect LGESS rather than rare leiomyoma variants, reflecting indolent but invasive pathological behaviors of LGESS.

Compared to leiomyosarcoma, less is known about the added value of DWI and ADC values in differentiation of LGESS from leiomyoma. Li et al. reported that 12 of 13 LGESS cases showed high SI relative to uterine endometrium and skeletal muscle, except one case with iso SI^18^. Similar to their results, all LGESS cases in our study showed iso/high SI to sciatic nerve on DWI. Considering that most rare leiomyoma variants also showed high/iso SI (88%/90% for R1/R2, Table 2), DWI is not expected to be helpful to differentiate LGESS from rare leiomyoma variants. However, with its high sensitivity for detecting LGESS, DWI can be a reliable tool to screen out LGESS in preoperative workup, especially for minimally invasive treatments. Additionally, LGESS results obtained for this study showed significantly lower ADC/ normalized ADC values than those of rare leiomyoma variants (*p* ≤ 0.02, Table 2). These quantitative features might improve diagnostic confidence, providing information about the cellular density of tumors^13^. Poor CCCs of ADC/ normalized ADC values might be because of the heterogeneity of LGESS and rare leiomyoma variants. To avoid cystic/necrotic/hemorrhagic areas, ADC values were obtained from three representative regions of interest placed on solid parts by the two readers independently, which could be less objective. Optimal methods to obtain ADC values with high CCC should be explored further.

The pre-defined MRI system showed very good diagnostic performance with high sensitivity (95% for both readers) and NPV (97% for both readers). The system based on qualitative assessments would be intuitive for radiologists to use, although it could be subjective. After dichotomizing the Likert scale (1–3 vs 4 & 5), the inter-rater agreement was improved (κ = 0.78). The system would pick up LGESS from leiomyoma and provide information leading to prevention of misdiagnosis of LGESS and treatment optimization. Its relatively low specificity (67%/69% for R1/R2) and PPV (60%/62% for R1/R2) might be because the three T2WI features (worm-like nodular extension, intra-tumoral low SI bands, and low SI rim) could be also observed in some of rare leiomyoma variants (Supplemental Table 2). Addition of other statistically significant T2WI features (such as speckled appearance) and of qualitative/quantitative evaluation of ADC map might improve the diagnostic performance of MRI.

It is noteworthy that one case of LGESS predominantly located in the endometrial cavity was not diagnosed correctly in both readers’ evaluation of the pre-defined MRI system (Fig. 3). All previously reported T2WI features are associated with infiltration patterns to the myometrium^15–17^. Koyama et al. described that low SI bands on T2WI was not apparent in the tumor confined to the endometrium^16^. LGESS with a very small part of myometrial infiltration can be a pitfall of the pre-defined MRI system; it might be difficult to differentiate from submucosal leiomyoma. In such a case, pretreatment biopsy represents an option when a tumor shows high SI on DWI with a low ADC value.

Our study has several limitations. First, the number of patients with LGESS was relatively small. Considering its extreme rarity, we conducted a multicenter study, and it included the largest number of LGESS patients in the previous studies of MRI features. Second, this retrospective study was conducted without validation. Selection bias should be taken into consideration. An external and/or prospective validation with more numerous patients must be done to introduce our results to clinical preoperative workup safely, especially for minimally invasive surgery. Third, the MRI protocols of respective institutions were diverse. They can affect evaluations of some features such as the DWI and ADC map. Regarding ADC values of tumors, we calculated normalized ADC values to reduce the bias. Fourth, the control group enrolled patients only from the two institutions and the ratio of LGESS and rare leiomyoma variants would be different from that in clinical settings. Also, the control group was heterogeneous and differentiations between LGESS and respective leiomyoma variants were not performed. The control group did not include other tumors mimicking LGESS, such as usual leiomyoma with atypical MRI findings, other leiomyoma variants, other uterine sarcoma, or endometrial cancer. Fifth, considering the rarity of LGESS, disease prevalence and a grade of suspicion would have influence on the results of the MRI systems. The diagnostic system would be appropriate to use in the pretreatment approach of cases with high suspicion while evaluating MRI rather than a screening approach.

In conclusion, we presented characteristic MRI features of LGESS in comparison to those of rare leiomyoma variants. We also presented the MRI diagnostic system for LGESS with very good diagnostic performance. MRI can be a reliable tool for optimization of treatment strategies for uterine tumor, avoiding inappropriate less-invasive treatment options.

## Supplementary Information

Supplementary Figures.

Supplementary Table S1.

Supplementary Table S2.
