# Supplementary material for: Differentiation of uterine low-grade endometrial stromal sarcoma from rare leiomyoma variants by magnetic resonance imaging
Source: Sci Rep. 2021 Sep 27;11:19124. doi: 10.1038/s41598-021-98473-z (PMC8476551; doi:10.1038/s41598-021-98473-z)
Supplement: Supplementary file 1 — Supplementary Figures. [file 41598_2021_98473_MOESM1_ESM.pptx]

## Slide 1
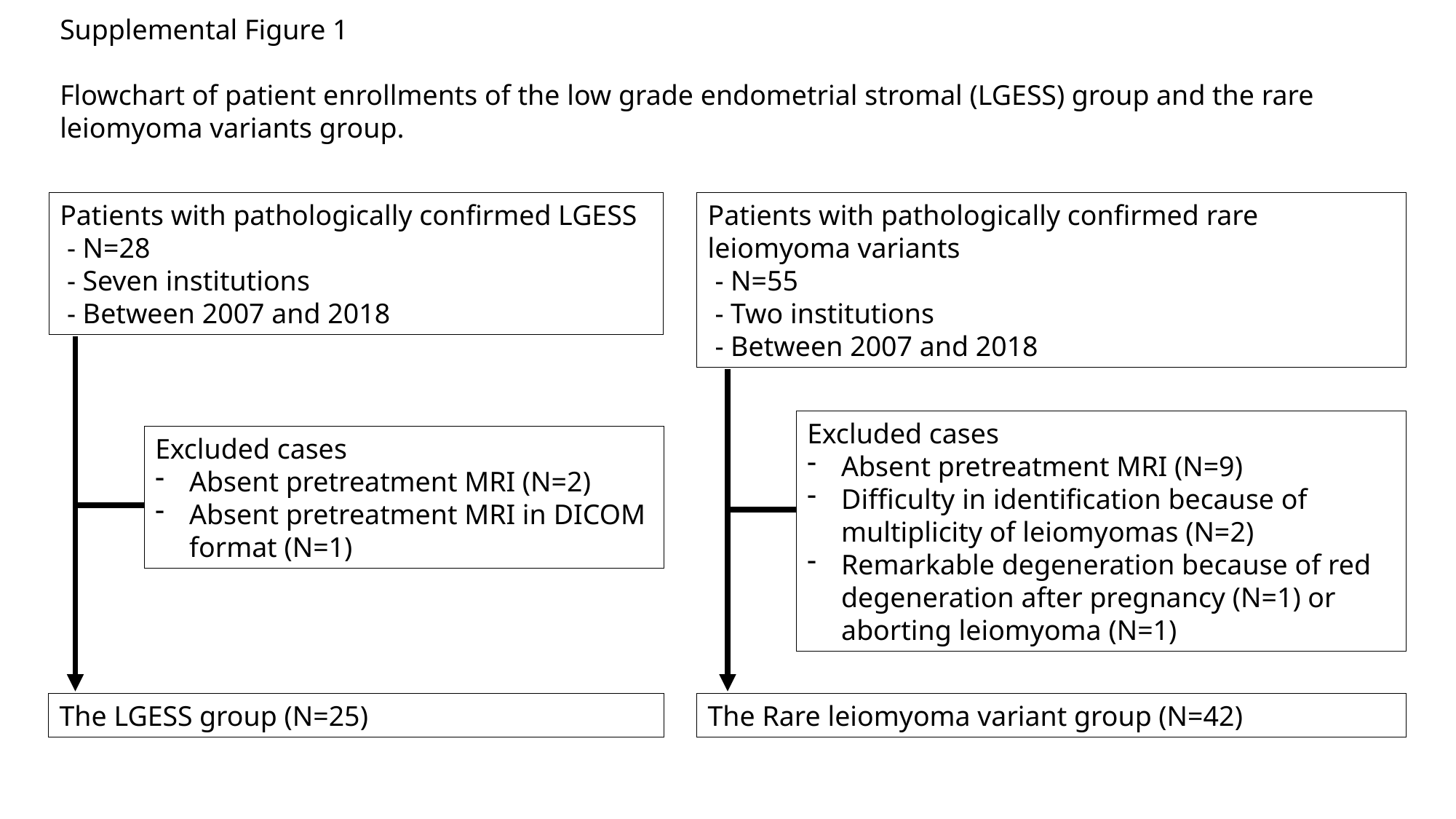

Supplemental Figure 1
Flowchart of patient enrollments of the low grade endometrial stromal (LGESS) group and the rare leiomyoma variants group.
Patients with pathologically confirmed LGESS
 - N=28
 - Seven institutions
 - Between 2007 and 2018
Patients with pathologically confirmed rare leiomyoma variants
 - N=55
 - Two institutions
 - Between 2007 and 2018
Excluded cases
Absent pretreatment MRI (N=9)
Difficulty in identification because of multiplicity of leiomyomas (N=2)
Remarkable degeneration because of red degeneration after pregnancy (N=1) or aborting leiomyoma (N=1)
Excluded cases
Absent pretreatment MRI (N=2)
Absent pretreatment MRI in DICOM format (N=1)
The LGESS group (N=25)
The Rare leiomyoma variant group (N=42)

## Slide 2
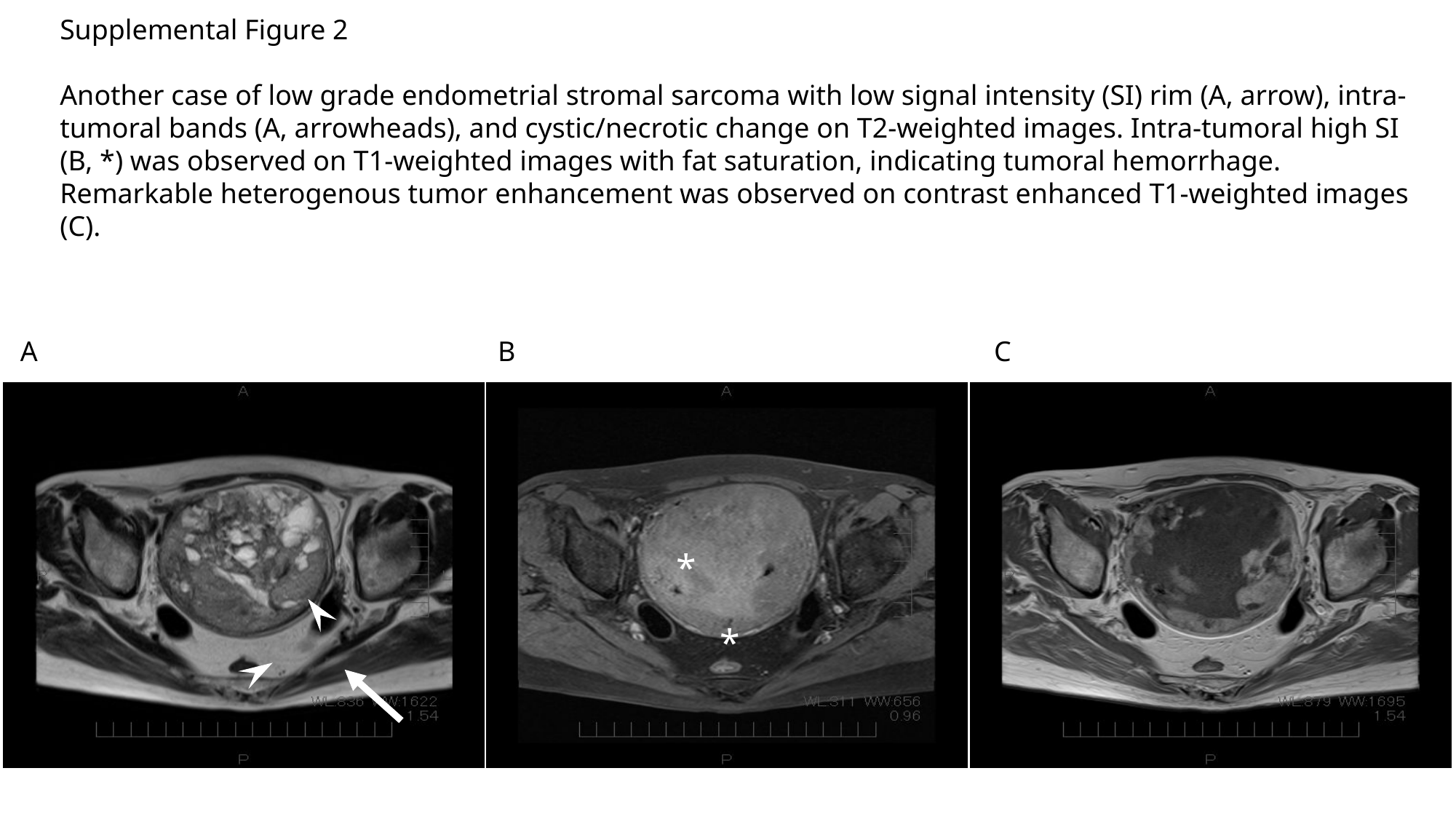

Supplemental Figure 2
Another case of low grade endometrial stromal sarcoma with low signal intensity (SI) rim (A, arrow), intra-tumoral bands (A, arrowheads), and cystic/necrotic change on T2-weighted images. Intra-tumoral high SI (B, *) was observed on T1-weighted images with fat saturation, indicating tumoral hemorrhage. Remarkable heterogenous tumor enhancement was observed on contrast enhanced T1-weighted images (C).
A
B
C
*
*

## Slide 3
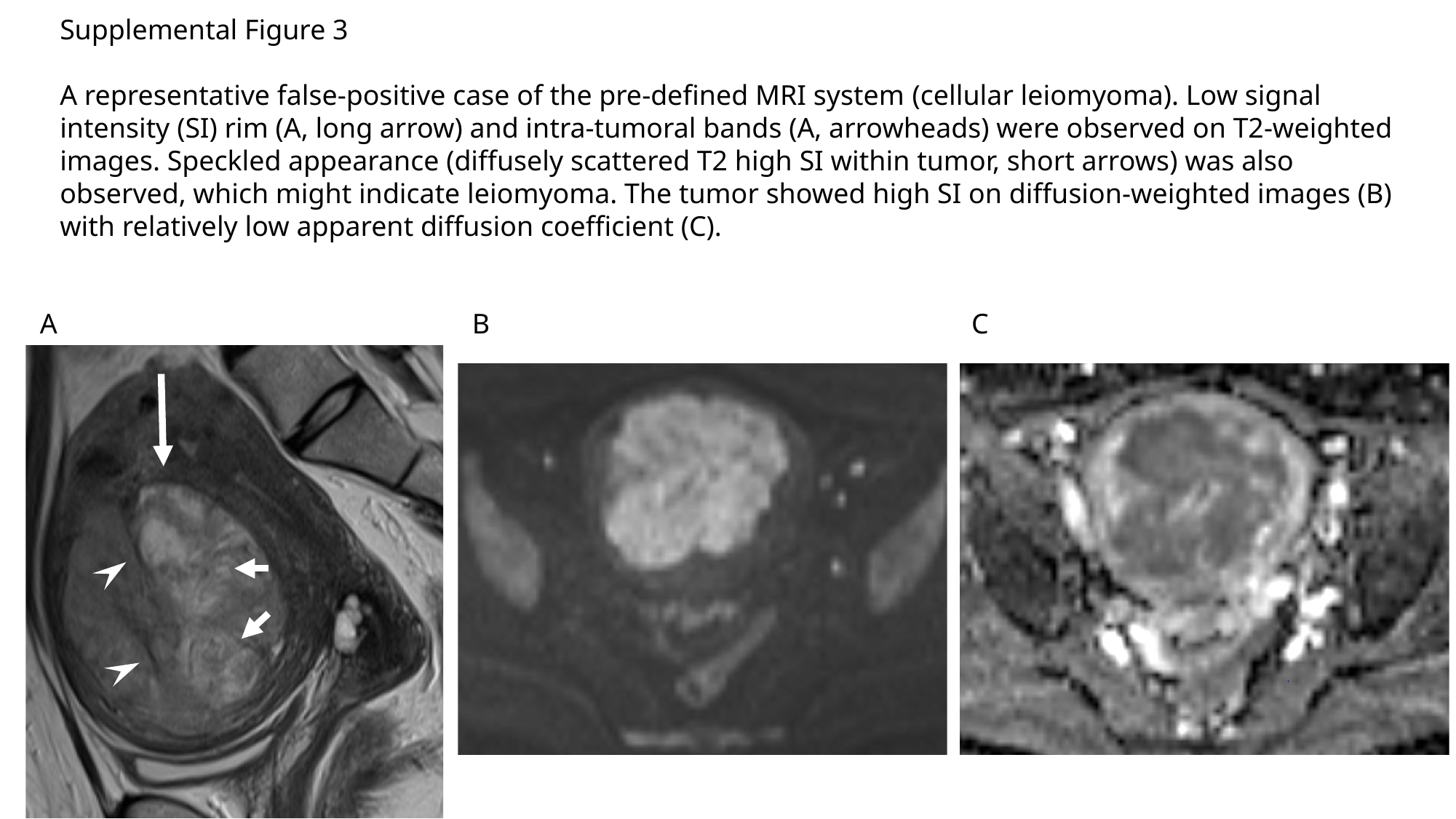

Supplemental Figure 3
A representative false-positive case of the pre-defined MRI system (cellular leiomyoma). Low signal intensity (SI) rim (A, long arrow) and intra-tumoral bands (A, arrowheads) were observed on T2-weighted images. Speckled appearance (diffusely scattered T2 high SI within tumor, short arrows) was also observed, which might indicate leiomyoma. The tumor showed high SI on diffusion-weighted images (B) with relatively low apparent diffusion coefficient (C).
A
B
C

## Slide 4
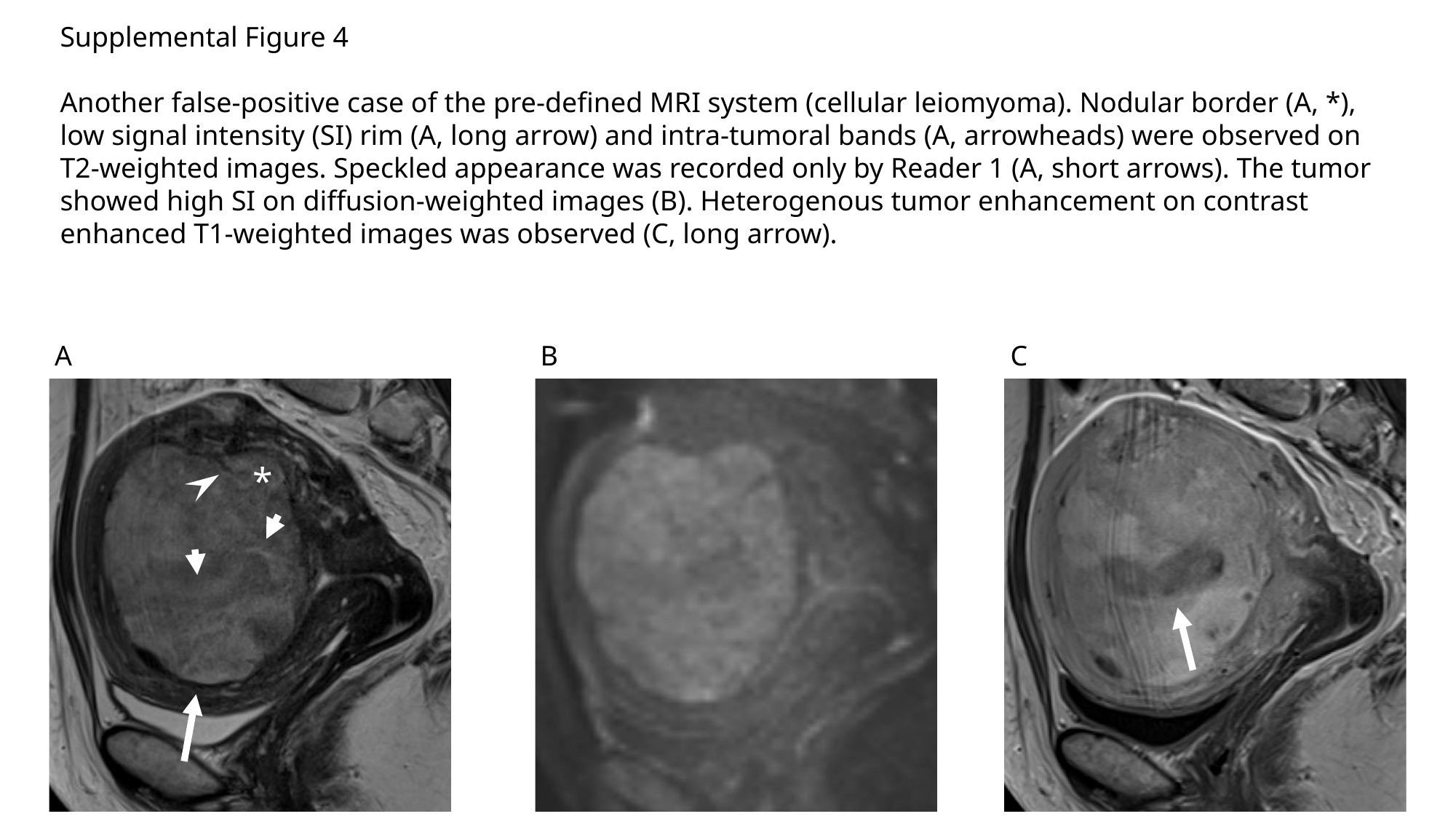

Supplemental Figure 4
Another false-positive case of the pre-defined MRI system (cellular leiomyoma). Nodular border (A, *), low signal intensity (SI) rim (A, long arrow) and intra-tumoral bands (A, arrowheads) were observed on T2-weighted images. Speckled appearance was recorded only by Reader 1 (A, short arrows). The tumor showed high SI on diffusion-weighted images (B). Heterogenous tumor enhancement on contrast enhanced T1-weighted images was observed (C, long arrow).
A
B
C
*
